# Differential proteomic analysis of virus-enriched fractions obtained from plasma pools of patients with dengue fever or severe dengue

**DOI:** 10.1186/s12879-015-1271-7

**Published:** 2015-11-14

**Authors:** Romain Fragnoud, Marie Flamand, Frederic Reynier, Philippe Buchy, Vasna Duong, Alexandre Pachot, Glaucia Paranhos-Baccala, Frederic Bedin

**Affiliations:** MD3 Department, BioMerieux SA, 69280 Marcy l’Etoile, France; UMR 3569 de Virologie Structurale, Institut Pasteur, 75015 Paris, France; BioASTER, 60007 Lyon, France; Unité de Virologie, Institut Pasteur Phnom-Penh, Phnom-Penh, Cambodia; Emerging Pathogens Laboratory Fondation Merieux, 69007 Lyon, France; MD3 & NAD Departments, BioMerieux SA, 69280 Marcy l’Etoile, France

**Keywords:** Dengue, Severity prognosis, Proteomics

## Abstract

**Background:**

Dengue is the most widespread mosquito-borne viral disease of public health concern. In some patients, endothelial cell and platelet dysfunction lead to life-threatening hemorrhagic dengue fever or dengue shock syndrome. Prognostication of disease severity is urgently required to improve patient management. The pathogenesis of severe dengue has not been fully elucidated, and the role of host proteins associated with viral particles has received little exploration.

**Methods:**

The proteomes of virion-enriched fractions purified from plasma pools of patients with dengue fever or severe dengue were compared. Virions were purified by ultracentrifugation combined with a water-insoluble polyelectrolyte-based technique. Following in-gel hydrolysis, peptides were analyzed by nano-liquid chromatography coupled to ion trap mass spectrometry and identified using data libraries.

**Results:**

Both dengue fever and severe dengue viral-enriched fractions contained identifiable viral envelope proteins and host cellular proteins. Canonical pathway analysis revealed the identified host proteins are mainly involved in the coagulation cascade, complement pathway or acute phase response signaling pathway. Some host proteins were over- or under-represented in plasma from patients with severe dengue compared to patients with dengue fever. ELISAs were used to validate differential expression of a selection of identified host proteins in individual plasma samples of patients with dengue fever compared to patients with severe dengue. Among 22 host proteins tested, two could differentiate between dengue fever and severe dengue in two independent cohorts (olfactomedin-4: area under the curve (AUC), 0.958; and platelet factor-4: AUC, 0.836).

**Conclusion:**

A novel technique of virion-enrichment from plasma has allowed to identify two host proteins that have prognostic value for classifying patients with acute dengue who are more likely to develop a severe dengue. The impact of these host proteins on pathogenicity and disease outcome are discussed.

**Electronic supplementary material:**

The online version of this article (doi:10.1186/s12879-015-1271-7) contains supplementary material, which is available to authorized users.

## Background

Infection by one of the four serotypes of the dengue virus (DV), a member of the *Flaviviridae* family, can cause a wide spectrum of clinical manifestations. Although the majority of symptomatic patients develop a febrile illness known as dengue fever (DF) with non-specific symptoms such as headache, fever or myalgia, around 10 % of patients develop a more severe form of disease, severe dengue (SD), that may include plasma leakage, severe hemorrhage and organ failure [1].

The DV genome is a positive single-stranded RNA molecule encoding a polyprotein that is processed into three structural proteins (the capsid protein C, the membrane protein M, and the envelope proteins E) and seven non-structural (NS) proteins involved in replication and pathogenicity [2]. DV enters target cells via receptor-mediated endocytosis and traffics via the endosome, where the acidic environment triggers fusion of viral and host cell membranes. Once within the target cell, the virus manipulates the host cell membrane to create an optimal environment for the assembly of its replication complex and subsequent RNA amplification [3]. Virion assembly occurs on the surface of the endoplasmic reticulum (ER), followed by budding of an immature particle into the ER lumen. The immature virion is then transported to the trans-Golgi network, matured via proteolytic cleavage, and finally released by exocytosis into the extracellular medium.

No specific antiviral treatment against DV currently exists. The available therapies are symptomatic and are administered to control the clinical manifestations. The ability to diagnose DV infection at an early stage and successful prognosis of the resulting complications of dengue are urgently required to improve the management of patients. It is possible that the identification of proteins specifically present in plasma before the onset of severe symptoms may ultimately lead to the discovery of new prognostic biomarkers.

Despite an incomplete understanding of the mechanisms of pathogenicity, several hypotheses have been formulated to explain the disease process in patients infected with DV. However, the lack of animal models capable of reproducing the features of human disease has hampered the identification of reliable parameters and indicators to explain or predict the development of SD.

A number of host immune components, especially antibodies, are associated with the pathogenicity of viral infections. Such mechanisms include antibody-dependent enhancement of a secondary infection or cross-reactivity with proteins such as endothelial cell or coagulation proteins [4–8]. Other immune components, including memory T-cells, innate immunity effectors and complement factors have been shown to modulate the outcomes [9, 10]. The DV non-structural protein 1 (NS1) may also play a major pathogenic role, as it interacts with host complement proteins [11, 12].

Numerous studies have investigated associations between altered levels of circulating cytokines/chemokines and dengue severity [13, 14].

Alternatively, as there is a strong biological rationale for investigating markers implicated in vascular pathologies, many markers such as the soluble intercellular adhesion molecule-1 (sICAM-1), the soluble vascular cell adhesion molecule-1 (VCAM-1), the e-selectin or the thrombomodulin have been identified and seemed to correlate with disease severity [15].

Unfortunately, no clear consensus has emerged from these studies. Predicting outcome in dengue remains challenging, and the search for robust markers remains crucial.

Previous studies have demonstrated patients with SD have higher viremia than patients with DF [9, 16, 17]. Additionally, reports from Cuba and Australia have suggested that circulating DV may become more virulent through passage in successive hosts during an epidemic [18–21].

As the virus cycle and the virus pathogenicity are strongly linked with the host metabolism, it is assumed that host proteins interacting with virions are a reflection of pathological status of the patient.

Therefore, in the present study, a DV-enrichment procedure combining sucrose gradient ultracentrifugation and a polymer-based technique was developed for differential proteomic analysis of plasma pools from patients with DF and SD by liquid chromatography coupled with tandem mass spectrometry (LC-MS/MS). The objective was to identify the viral proteins and the host proteins incorporated into virions, and also the host proteins that interact/are associated with virions. The identified markers were validated by quantitative ELISAs using samples from dengue patients from two different geographical regions. The possible role of the co-identified host proteins in disease pathogenicity and the potential of these proteins as fingerprints of disease severity in patients infected with DV are discussed.

## Methods

### Plasma specimens

Plasma samples were provided by the Universidad Industrial de Santander, Bucaramanga, Colombia and the Institut Pasteur, Phnom-Penh, Cambodia. Samples were collected from dengue patients as part of retrospective (Colombia) or prospective study (Cambodia). Both studies were reviewed and approved by the local Medical Ethics Committees (Universidad Industrial de Santander, Colombia; National Ethic Committee, Cambodia) and performed in compliance with the ethical standards set out by the Declaration of Helsinki. All patient plasma samples were anonymized after a physical examination and obtaining informed consent. Dr Villar-Centeno (Universidad Industrial de Santander, Bucaramanga, Colombia) and Dr Philippe Buchy (Institut Pasteur, Phnom-Penh, Cambodia) granted the authors permission to use the samples.

All samples were collected between the onset of symptoms and defervescence. The cases were classified as primary or secondary infections by the physician based on hemagglutination assays performed on different DV serotypes and on Japanese encephalitis virus. The serotype and the copy number were determined by real-time quantitative RT-PCR (qRT-PCR). DV-negative plasma specimens from healthy donors were obtained from the French National Blood Bank (Etablissement Français du Sang, Lyon, France). Dengue samples were tested for the presence of the viral NS1 protein using the Platelia™ ELISA (BioRad, Marnes-la-Coquette, France) following the manufacturer’s instructions.

### Quantification of DV RNA

RNA was extracted from the plasma using the QIAmp Viral RNA kit (Qiagen, Hilden, Germany). Viremia was measured using a qRTPCR kit (PrimerDesign Southampton, UK) according to the manufacturer’s instructions. QRT-PCR was performed in a final volume of 20 μl, containing 5 μl of extracted RNA, 10 μl of 2x Precision OneStepTM qRT-PCR MasterMix, and 1 μl of Dengue Primer/Probe mix. Assays were carried out using a LightCycler® 1.2 (Roche Applied Science, Bâle, Switzerland) using the OneStep amplification protocol recommended by the manufacturer.

### Cell culture and infection

HepG2 cells (ATCC HB-8065) were cultivated at 37 °C in 5 % CO_2_ in DMEM supplemented with 10 % decomplemented fetal calf serum, 5 × 10^4^ IU Penicillin, 50 mg streptomycin and 10 mM *L-*glutamine (Invitrogen, Paisley, UK). The cells were infected as previously described [22] with a serotype 3 DV (DV3, strain D78-878 Thailand) graciously provided by Dr V. Barban (Sanofi-Pasteur, Lyon, France). Sub-confluent HepG2 cell cultures (approx. 10^7^ cells/75 cm^3^ flask) were incubated with virus diluted in serum-free culture medium at various multiplicities of infection (MOIs) for 90 min, the supernatant was removed, the cells were washed once with PBS (Invitrogen) and 10 mL of fresh complete medium was added to the cells. After 5 days of culture, the supernatant was harvested and clarified by centrifugation at 10,000 g for 5 min at 4 °C.

### Denaturing polyacrylamide gel electrophoresis, Western blotting and silver-staining

Following denaturation in SDS sample-buffer (Novex Invitrogen, Paisley, UK) at 37 °C and denaturing polyacrylamide gel electrophoresis (PAGE) on 4–12 % polyacrylamide gels in SDS-MOPS buffer (NuPage Invitrogen), samples were electro-transferred to PVDF membranes (Millipore, Billerica, MT, USA) in 10 % CAPS-10 % methanol buffer, blocked in TBS-0.1 % Tween 20–5 % skimmed dried milk (Régilait, Macon, France), incubated with anti-E monoclonal antibody (diluted to 1 μg/mL; BioMerieux, Marcy-l’Etoile, France) followed by horseradish peroxidase-labeled conjugate (diluted at 0.1 μg/mL; P.A.R.I.S., Compiegne, France) for 1 h each at room temperature (RT). After washing with TBS-0.1 % Tween 20, the proteins were revealed using the SuperSignal West Dura Kit (Thermo Scientific, Rockford, IL, USA) and imaged using the VersaDoc™ Imaging System (BioRad, Hercules, CA, USA).

Alternatively, the gels were silver-stained after electrophoresis using the SilverXpress Kit (Life Technologies, Paisley, UK). Densitometry analysis was carried out using Quantity-One software (BioRad).

### Preparation of DV-enriched fractions

Five milliliters of pre-clarified plasma pools (5000 g/10 min/4 °C; cf. Table 1) or 5 ml of pre-clarified cell culture supernatant were centrifuged for 2 h at 200,000 g using a Beckman SW41 rotor in an Optima L90 ultracentrifuge (Beckman, Fullerton, CA, USA). After centrifugation, the pellet was dissolved in a small volume of cold PBS (Euromedex, Souffelweyersheim, France), loaded on a discontinuous sucrose gradient constituted of 5 ml of 60 % sucrose in PBS (*w/w*) and 5 ml of 20 % sucrose in PBS (*w/w*), and centrifuged for 2 h at 200,000 g. The fraction containing virions, located at the interface of the two sucrose solutions, was collected, diluted ten-fold in cold PBS, centrifuged for 2 h at 200,000 g and the pellet was resuspended in 200 μl of cold PBS. All centrifugation steps were performed at 4 °C.

**Table 1:** Characteristics of the Colombian patients whose plasma samples were pooled to prepare the viral-enriched fractions

	DF Pool	SD Pool
Number of patients	8	7
Age, mean (years)	26.8	33.7
Male/female ratio	6/2	3/4
Day of sampling, mean	3.3	2.7
Secondary infections	8 (100 %)	7 (100 %)
Viral titer (copies/mL)	4.05 10^6^	4.13 10^7^
NS1 positive	8 (100 %)	8 (100 %)
DV serotype 2	2 (25 %)	3 (42.8 %)
DV serotype 3	6 (75 %)	4 (57.1 %)

Data is shown as mean or number (percentage)

Ultracentrifugation was complemented by additional purification steps using Viraffinty™ (BiotechSupportGroup, Monmouth, USA), a water insoluble elastomeric polyelectrolyte developed for the capture and recovery of viruses [23], according to the manufacturer’s instructions. Briefly, 200 μl of MN buffer (60 mM MES pH 6.5, 150 mM NaCl) and 100 μL of Viraffinity™ were added to the resuspended pellet obtained after ultracentrifugation. The mixture was incubated for 5 min at RT and centrifuged at 1000 g for 10 min. The supernatant was discarded and the pellet was rinsed three times with MN buffer. Finally, the viral fraction was separated from the polymer by heating for 5 min at 70 °C in SDS-buffer (Novex Invitrogen). After a final centrifugation step at 1000 g, the supernatant was harvested and stored at −80 °C for immunoblotting and LC-MS/MS analysis.

### In-gel digestion and LC-MS/MS analysis

The following experiments were conducted by the EDyP laboratory (EDyP-Service, CEA Grenoble, France). The virion-enriched preparation was loaded on a 10 % polyacrylamide gel and electrophoresed until all proteins entered the gel. The band containing the proteins was manually excised, washed three times in buffer containing 50 % acetonitrile and dried using 100 % acetonitrile. The gel piece was rehydrated with 7 % H_2_O_2_ for 15 min in the dark, washed, and incubated in a modified sequencing-grade trypsin solution (Promega, Madison WI, USA) in 25 mM NH_4_HCO_3_ overnight at 37 °C. Peptides were extracted from the gel band via three sequential extraction steps of 15 min each with 30 μl of 50 % acetonitrile, 30 μl of 5 % formic acid and 30 μl of 100 % acetonitrile. The extracts were pooled, vacuum dried and resuspended in a solution containing 5 % acetonitrile and 0.1 % trifluoroacetic acid. After quantity normalization, the peptides were analyzed by nanoliquid chromatography coupled to tandem mass spectrometry (nanoLC–MS/MS, Ultimate 3000; Dionex, Sunnyvale, CA and LTQ-Orbitrap VelosPro; ThermoFisher Scientific, Rockford, IL, USA). Data was acquired using Xcalibur software (ThermoFisher Scientific) and processed automatically using Mascot Daemon software (version 2.2; Matrix Science, Boston, MA, USA).

Proteins with a Mascot score higher than 40 (*P* < 0.05) were selected for further analysis [24]. The Mascot score is a measure of the reliability of identification: the higher the score, the better the identification. In order to eliminate false matches and incorrect protein identification, consecutive searches against the concatenated Swiss-Prot and Trembl_decoy databases (versions 57.6 and 40.6 decoy database, respectively; Homo sapiens taxonomy, 164,620 entries) were performed for each sample using Mascot 2.3 software (Matrix Science). IRMa software [25] was used to filter the results to achieve a false positive rate lower than 1 %. In-gel digestion and LC-MS/MS analysis was performed twice for each sample.

### Canonical pathway analyses

Ingenuity Pathways Analysis (IPA) software (Ingenuity Systems, Redwood City, CA, USA) was used to investigate the interactions among all of the host proteins identified. Interactive pathways were generated to observe the potential direct and indirect relationships among the proteins that were differentially expressed in the DF and SD samples.

### Quantitative ELISA

Commercial ELISAs (USCN, Wuhan, China) targeting potential severity markers were used to measure in duplicate the levels of the proteins in individual DF and SD plasma specimen, using the protocols recommended by the manufacturer. For each test, a standard curve was established by serial dilution of the calibrator provided in the kit to determine the protein concentration. The optical density values were determined at 450 nm using a microplate reader (Eon; BioTek, Vinooski, VT, USA). Statistical analysis (Mann–Whitney *U* test, Chi-square test) and Receiver Operating Curve analysis were performed using GraphPad Prism V.4.03 software (GraphPad Software, San Diego, CA, USA). The Mann–Whitney and the Chi-square tests were used to examine differences in demographic and clinical characteristics between patients and to assess potential confounding variables. Comparisons of continuous variables were performed using Mann–Whitney *U* test that can be applied on unknown distributions for two small sets of observation (*n* < 30). Comparisons of proportion were performed using Chi-square test.

*P* < 0.05 was considered significant [26].

## Results

### Preparation of plasma pools for purification

Plasma obtained from patients with DF or SD were pooled to create two samples (Table 1); all of the plasma samples used for this step came from Colombian patients who had secondary DV serotype 2 or 3 infections, and were collected between the onset of symptoms and the development of severe symptoms [27]. Classification of the infections as secondary infections and the degree of severity were based on review of the patients’ medical records. In these medical records, the estimation of the severity was based on the WHO criteria of 1997 [1]. DF and DHF grade I (Dengue Haemorrhagic Fever, minor haemorrhages) were considered as classicdengues (not severe). DHF grade III/IV (DSS, Dengue Shock Syndrome) were considered as severe dengues. Each medical records compiled details on platelet and blood cell counts, transaminase levels, the presence/absence of warning signs (persistent vomiting, abdominal pain…), haemorrhagic signs (petechiae, ecchymosis, epistaxis…) or shock signs (cold extremities, cyanosis…) that help to the patients classification by the physician.

The proportion of samples from male patients was higher for the DF pool (75 %) than the SD pool (43 %). The age of the patients (mean, 30 years) and the number of days the samples were collected after the onset of symptoms (mean, approximately 3 days) were similar between the two groups. No comorbidity was reported for any patient in either group.

Each of the individual plasma samples was confirmed as NS1-positive. The viremia of each pool was estimated using a commercial qRT-PCR kit. All patients were positive for DV, indicating all samples were collected during the acute phase of disease. The average number of RNA copies per mL was 4.05 × 10^6^ and 4.13 × 10^7^ for the samples in the DF and the SD pools, respectively.

### Isolation of the viral-enriched fraction from the plasma of patients infected with DV

A technique based on ultracentifugation (Step 1) followed by concentration using a commercial water-insoluble elastomeric polyelectrolyte specially engineered for the capture and recovery of viruses (Viraffinity™; Step 2), was created to obtain a fraction of plasma enriched with DV particles.

**Fig. 1:**
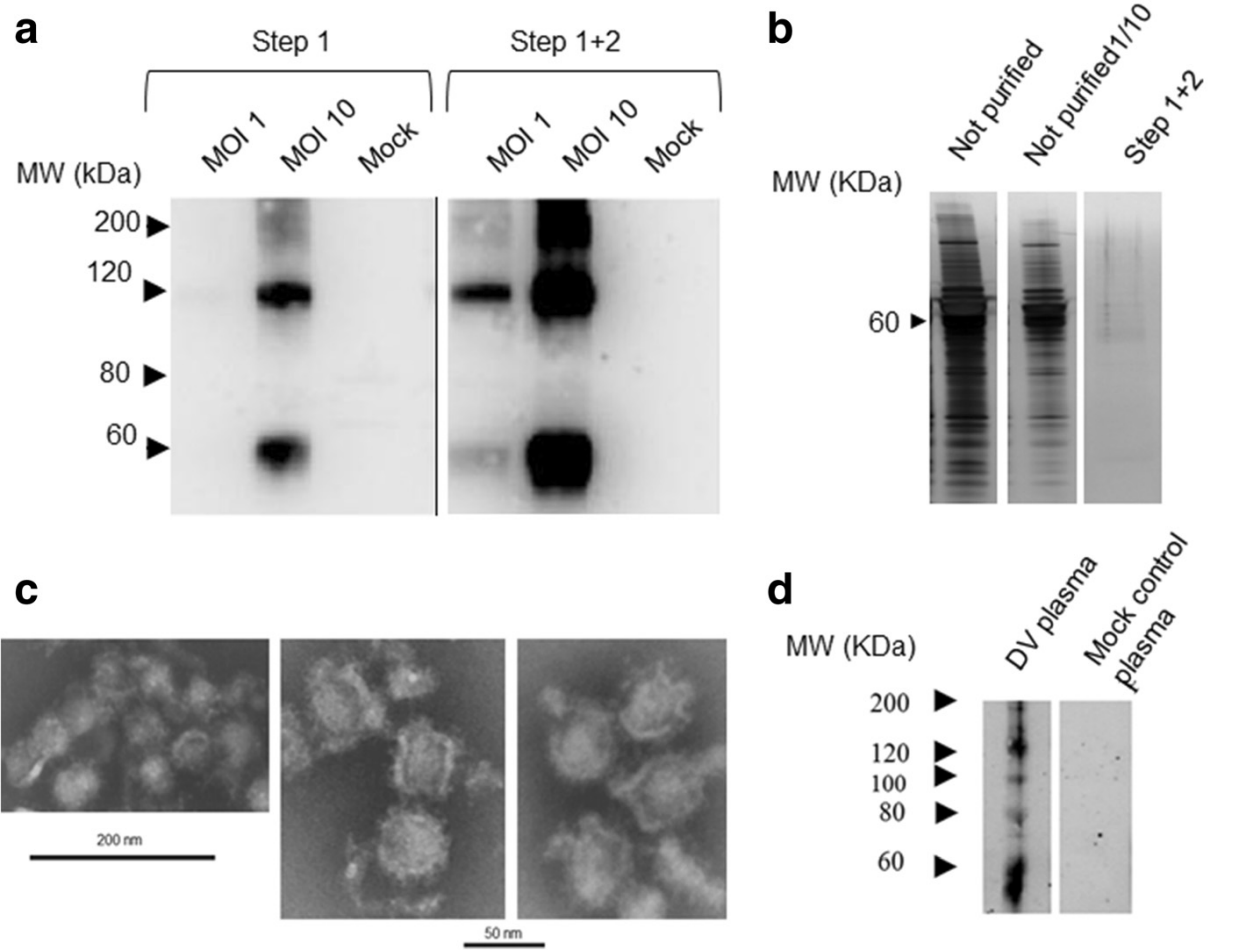
Characterization of the viral-enriched fraction of DV-infected HepG2 supernatant or plasma obtained from patients with dengue. **a** After infection with DV at a MOI of 1 or 10, HepG2 cell culture supernatants were purified by ultracentrifugation (Step 1) followed by the use of Viraffinity™ (Step 1 + 2). **b** Silver-staining assessment of the protein complexity of DV-infected HepG2 cell culture supernatant before and after purification; not diluted (lane “not purified”), 1/10 diluted (lane “not purified 1/10”) DV-infected cell supernatant before purification or the same sample as shown in lane 1 after purification (lane “Step 1 + 2”) were separated by denaturing polyacrylamide gel electrophoresis and silver-stained. **c** Electron microscopy of DV-infected HepG2 culture supernatant after ultracentrifugation. Grids were negatively stained using uranyl acetate. Bars: 50 nm, 200 nm. **d** Representative Western blotting analysis of plasma pools obtained from patients with dengue after purification by ultracentrifugation and Viraffinity™ using an anti-E monoclonal antibody. MW: molecular mass

Initially, this technique was developed using the cell culture supernatant of HepG2 cells harvested five days after infection with DV at a MOI of 1 or 10. The samples obtained after each step of the purification process (Step 1 and Step 1 + 2) were analyzed by Western blotting using an anti-E monoclonal antibody (Fig. 1a). A positive signal corresponding to monomeric (60 kDa) and dimeric E protein (120 KDa) was observed in the DV-infected samples, and became more intense after the Viraffinity™ step (Fig. 1a, Step 1 + 2). The purity of the Step 1 + 2 sample was assessed by electrophoresis using denaturing PAGE and silver-staining (Fig. 1b). Bovine serum albumin (BSA), an intense protein band of approximately 66 kDa was observed in the sample before purification (“not purified” lanes); however, this band was almost absent from the final purified sample (“Step 1 + 2” lane). Only a small number of bands were detected in the purified sample. Densitometry indicated that the protein complexity of the purified samples was reduced by roughly 350-fold compared to the unpurified samples.

The presence of intact viral particles was also confirmed by electron microscopy, which was performed by the EZUS laboratory (Université Claude Bernard, Villeurbanne, France). After negative staining, virions could be visualized in the purified samples obtained from the supernatant of cells infected at a MOI of 10 (Fig. 1c). The viral membrane, including the E spikes, was visible. The spikes observed on the virion surface are probably due to the slightly basic pH (pH 7.5) of the PBS-buffered solutions used during centrifugation and electron microscopy. Virions tended to aggregate in clusters of 10–20 viral particles as seen in images taken at a lower magnification. Little background signal was observed around the virion clusters.

Following the two-step purification process, the MOI 10 sample was also analyzed by nano-LC MS/MS. All structural viral proteins were identified (Table 2). The E protein peptides were the most largely represented (21 peptides; 15 single peptides). The sequence coverage for the E protein represented 44 % of the entire protein. The protein M, which is part of the external viral layer, along with the E protein, was identified three times (3 single peptides) with coverage of more than 58 % of the protein sequence. The pr peptide and capsid were also identified (one peptide each).

**Table 2:** DV peptides identified by LC-MS/MS analysis of the purified supernatant of HepG2 infected with DV (MOI10)

Viral protein	Peptide sequence	Number of peptides detected	Sequence coverage (%)
Capsid C	EISNMLSIINK	1	11.00
Peptide pr	TASGINMCTLIAMDLGEMCDDTVTYK	1	28.57
	SVALAPHVGMGLDTR	1	
Membrane protein	VETWALR	1	58.67
M	HPGFTILALFLAHYIGTSLTQK	1	
	DFVEGLSGATWVDVVLEHGGCVTTMAK	1	
	NKPTLDIELQK	1	
	TEATQLATLR	1	
	CPTQGEAILPEEQDQNYVCK	2	
	GWGNGCGLFGK	2	
	QWFFDLPLPWTSGATTEAPTWNR	2	
	EVSETQHGTILIK	1	
Envelope protein	VEYKGEDAPCK	1	44.00
E	IPFSTEDGQGK	1	
	LITANPVVTK	1	
	KEEPVNIEAEPPFGESNIVIGIGDK	1	
	EEPVNIEAEPPFGESNIVIGIGDK	1	
	MFEATAR	1	
	MAILGDTAWDFGSVGGVLNSLGK	3	
	IGIGVLLTWIGLNSK	2	

In conclusion, combining ultracentrifugation with Viraffinity polymer yielded a fraction with a considerably reduced protein complexity that contained a large quantity of enveloped virions.

This technique was then applied to the pooled plasma samples obtained from the Colombian patients (Table 1). After purification, the preparations were analyzed by Western blotting using a monoclonal antibody directed against the E viral protein (Fig. 1d, SD pool as an example). Strong signals corresponding to monomeric (60 kDa) and dimeric E protein (120 kDa) were observed. The E protein was not detected in purified plasma obtained from healthy individuals, which was tested as a mock-control.

### Viral and host proteins identified by nano-LC-MS/MS in purified plasma pools

To identify the proteins present in the purified samples and to compare their composition, nano-LC-MS/MS was conducted on the purified DF and SD plasma pools. This experiment was also performed on the purified mock-control sample to determine the host background. The same quantity of total protein was analyzed for each sample. These experiments were performed independently twice.

Both viral and host peptides were identified in the purified pooled plasma samples from the patients with DF and SD. One peptide corresponding to the viral envelope protein was detected twice in the SD sample; this peptide (GWGNGCGLLFGK) was also identified in the purified DV-infected HepG2 supernatant (Table 2).

After removal of the mock-control background, the remaining peptides identified in the DF and SD samples were analyzed. In order to consolidate the results obtained by nano-LC MS/MS, only proteins for which the variance of the average number of peptides was lower than 25 % were selected [28]; 188 host proteins met this criterion. The SD/DF peptide ratio was calculated for these 188 proteins (see Additional file 1). The highest SD/DF ratio was obtained for C1s esterase (SD/DF peptide ratio = 4.14) and the vitamin K-dependent protein S (ratio = 4). The lowest ratio was obtained for beta-1 spectrin (ratio = 0.13). Six proteins were only identified in the SD sample; consequently, the SD/DF ratio could not be calculated for these proteins.

### Canonical pathway analysis

**Fig. 2:**
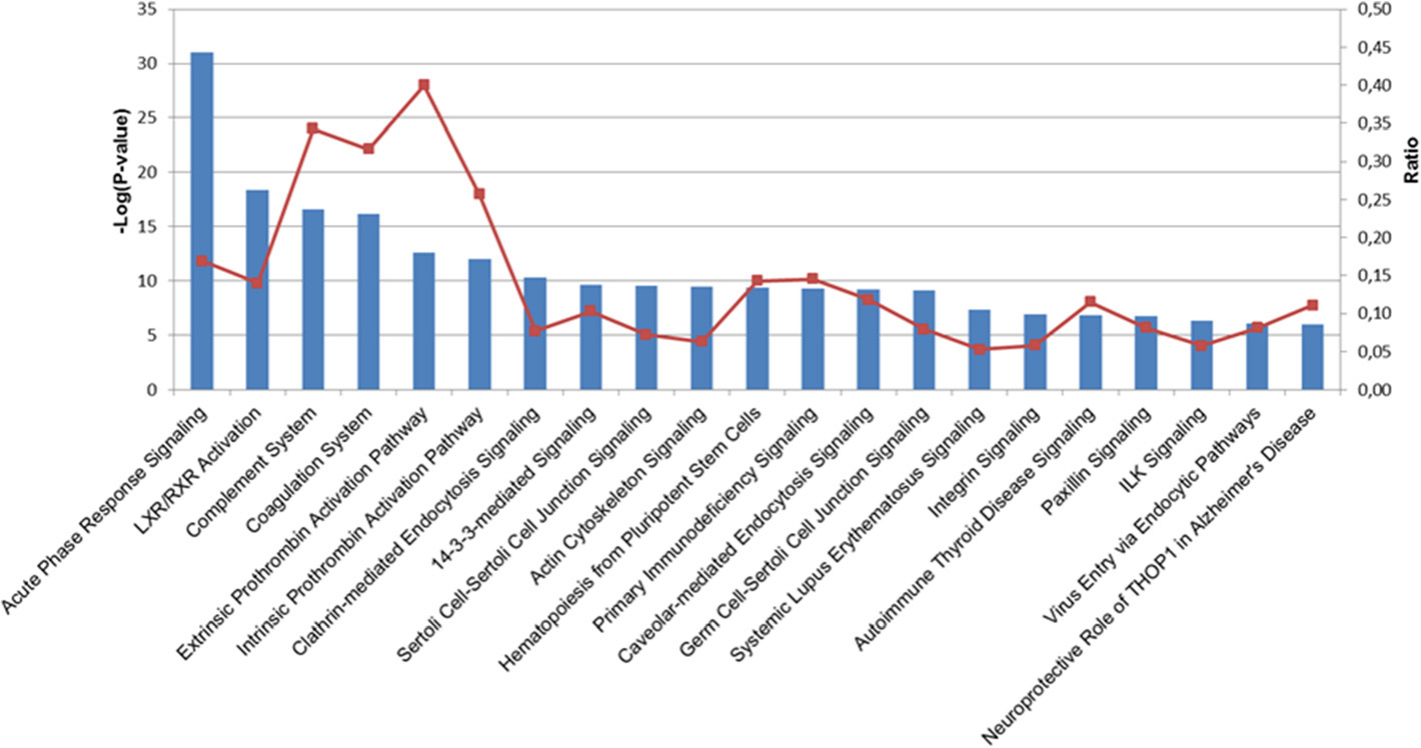
Ingenuity Pathway Analysis for host proteins identified in the viral-enriched plasma fraction of patients with dengue. Pathway classification according to canonical pathways was performed using IPA software. The *x*-axis represents the pathways identified. The *y*-axis (left) shows the − log of the *P*-value calculated using Fisher’s exact test. The ratio (*y*-axis, right) represented by the line is calculated as follows: number of proteins in a given pathway that meet the cutoff criteria divided by total number of proteins that make up that pathway

Ingenuity Pathway Analysis (IPA) was conducted to further elucidate the specific pathways associated with the identified host proteins. The specific location and function of each pathway was attributed by IPA for 174 of the 188 proteins; the most prominently represented pathways are illustrated in Fig. 2. The IPA diagram shows that the highest *P*-values were attributed to Acute Phase Response Signaling [−log(*p*-value), LP = 31], the LXR/RXR Activation Pathway (LP = 18.4), the Complement System (LP = 16.6), the Coagulation System (LP = 16.2) and the Extrinsic Prothrombin Activation Pathway (LP = 12.6). The *P*-values indicate the likelihood that the focus genes in a network are found in these pathways by random chance alone.

Although represented to a lesser degree, three pathways related to host proteins involved in the DV cell cycle, Clathrin-Mediated Endocytosis Signaling (LP = 10.3), Caveolar-Mediated Endocytosis Signaling (LP = 9.2) and the Virus Entry via Endocytic Pathway (LP = 6.05), were also identified. Other identified pathways, including Integrin (LP = 6.96) and Paxillin Signaling (LP = 6.75), correspond to proteins involved in cell-matrix interactions and cell-to-cell communication.

Figure 2 also illustrates the ratio of identified proteins for each canonical pathway. Higher proportions of proteins mapped to the Complement System (ratio = 0.34), Coagulation System (ratio = 0.32) and Extrinsic Prothrombin Activation Pathway (ratio = 0.4) than the other pathways (ratios between 0.16 and 0.05).

IPA software was also used to investigate possible interactions among all of the identified proteins and to assess the representation of the identified proteins in Acute Phase Response Signaling, the Complement System and the Coagulation System with Prothrombin Activation (see Additional file 2).

Twenty of the 50 (40 %) secreted proteins identified by IPA as part of the Acute Phase Response Signaling pathway, which is activated in macrophages and endothelial cells upon infection and inflammation, were over-represented in the pooled SD plasma sample compared to the pooled DF plasma sample. Thirteen Complement Component proteins were also over-represented in SD plasma, with high numbers of peptides identified for C1r and C1s in particular. Complement factor C8 and complement factor B (CFB), which are involved in the complement alternate pathway, were only identified in the pooled SD plasma sample. The C9 protein was enriched in the pooled DF plasma sample. The Extrinsic and Intrinsic Prothrombin Activation pathways are part of the Coagulation system. The majority of the proteins involved in the Fibrinogen/Fibrin cascade (i.e. 10 proteins out of 11) were over-represented in the SD sample compared to the DF sample.

Overall, the network analysis also provided evidence of strong links between these three pathways. The coagulation factor 2 (F2) and the C3 and C5 proteins are located at the interface of the Coagulation System and the Complement System (see Additional file 3).

### Validation of the proteins in individual plasma specimens using quantitative ELISAs

To validate the mass spectrometry data, the levels of selected host proteins were assessed by quantitative ELISA both in the virus-enriched fraction and in individual plasma samples from DF or SD patients.

For the ELISA, proteins with a SD/DF ratio higher than 1.3 or lower than 0.78 were selected (see Additional file 1); other criteria, such as the number of peptides identified in the SD sample and the availability of a commercial ELISA kit were also considered. Some proteins, such as ribosomal protein P2 (accession number P05387; ratio = 0.6) and histone H4 (accession number P62805; ratio = 0.4) were not deemed relevant enough for the study and were consequently not tested. The proteins readily found at high concentrations in plasma and known to be frequent contaminants in proteomic experiments, such as the immunoglobulin heavy chain (accession number P01779; ratio = 2.4), were not tested. Finally, a non-exhaustive list of 22 proteins to assay was established.

These 22 proteins were ceruloplasmin (CP), vitamin K-dependent protein S, complement factor properdin, antithrombin III, secretory component p85, complement factor C1r (C1r), complement factor C1s, angiotensin, factor 2, anti-factor VIII, serum amyloide P-component, olfactomedin-4 (OLFM4), thrombospondin, gelsolin, platelet factor 4 (PF4), complement factor C1q, complement factor B, moesin and complement factor C8. Multimerin-1, apolipoprotein B-100 and von Willebrand factor were also tested.

These 22 proteins were first quantified in the virus-enriched fraction of the individual plasma samples obtained from DF or SD patients of the Colombian cohort. The ELISA signal levels for these samples were close to background levels (purified mock-control sample); the results were not interpretable.

To investigate the potential interaction of these proteins with the virion, fresh plasma was incubated for various times with purified viral particles. The change of protein concentration before and after the incubation was assessed by ELISA. This experiment was performed for 4 proteins out of 22 (OLFM4, CP, C1R, PF4) and showed that no significant signal change was observed for these proteins, whatever the condition tested (data not shown). The virus preparation is probably not concentrated enough to induce a significant variation in host protein concentration. Furthermore, the plasmatic concentration of the four proteins is too high to be significantly affected by the interactions with the virus coated in the microplates.

**Fig. 3:**
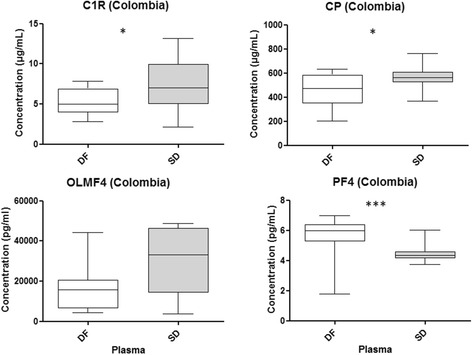
Assessment of the protein concentrations of C1r, CP, OLFM4 and PF4 in the individual plasma samples of Colombian patients using specific quantitative ELISAs. Each value corresponds to the mean of two independent experiments, each performed in duplicate. *: 0.01 < *p* < 0.05; **: 0.005 < *p* < 0.01; ***: *p* < 0.005

The ELISAs were then performed on the whole plasma samples obtained from Colombian patients (Table 3, Colombian patients). All patients had secondary infections associated with various DV serotypes (DV1, DV2 and DV3); all samples were collected between onset and defervescence (between days 2 and 5). The male/female sex ratio and age were similar between the groups of patients with DF or SD. The samples were confirmed positive for both DV by qRT-PCR and the viral NS1 protein using a commercial ELISA (NS1 Platelia™). No comorbidity was recorded in any patient. Among the 22 proteins tested, only CP, C1r, OLFM4 and PF4, had different plasma concentrations between DF and SD patients. The average concentration of C1r was higher for patients with SD than patients with DF (*P* = 0.049). Moreover, the variance between the two groups was significantly different (*P* = 0.019). The protein concentration of OLFM4 was considered higher for SD patients (*P* = 0.051). For CP, the difference between the two groups was significant (*P* = 0.045). The largest difference was observed for PF4 with a higher concentration for patients with DF (*P* < 0.001; Fig. 3).

**Table 3:** Clinical profiles of the patients whose plasma samples were analyzed by ELISA

	Colombian Patients			Cambodian Patients		
	DF (*n* = 15)	DS (*n* = 15)	*P*-value	DF (*n* = 23)	DS (*n* = 26)	*P*-value
Age, average ± SD (years)	29.3 ± 0.52	28.3 ± 0.5	ns	8.6 ± 0.38	7.3 ± 0.45	ns
% male	60 %	53 %	ns	56 %	35.6 %	ns
Sampling (day after onset)	3.06	2.37	ns	2.6	3.2	ns
Weight, average ± SD (Kg)	60.2 ± 8.6	50.3 ± 15.6	ns	20.48 ± 0.93	18.86 ± 0.98	ns
Total cholesterol (g/L)	1.44 ± 5.4	1.29 ± 10.1	0.03	1.28 ± 0.05	0.92 ± 0.046	<0.0001
Aspartate aminotransferase (IU/L)	90.2 ± 9.75	142.7 ± 29.8	0.001	133.3 ± 23.18	302.5 ± 50.7	0.0043
Alanine aminotransferase (IU/L)	69.7 ± 11.4	90.8 ± 24.5	ns	66.9 ± 13.4	106.3 ± 21.2	ns
Viral load (copy/mL)	4.05 × 10^6^ ± 3.5	4.1 × 10^7^ ± 5.22	0.006	7.1 10^8^ ± 6	2.06 10^8^ ± 2	ns
Positive Tourniquet test	8/15 (60 %)	10/15 (66 %)	ns	19/23 (82 %)	18/26 (82.6 %)	ns
Hepatomegaly on ultrasound	na	na	-	8/12 (66 %)	16/26 (61.5 %)	ns
Serotype	1-2-3	1-2-3	-	1	1	-
Secondary dengue infection	15 (100 %)	15 (100 %)	-	23 (100 %)	26 (100 %)	-
Comorbidity	0 (0 %)	0 (0 %)	-	0 (0 %)	0 (0 %)	-

*IU* international units, *ns* not significant, *na* not applicable

Chi-square or Mann–Whitney tests were used to analyze the differences between groups

To further confirm the relevance of C1r, CP, OLFM4 and PF4 as potential markers to differenciate DF and SD, plasma samples from patients with acute dengue from Cambodia, another dengue-endemic area, were tested. As for Colombian patients, the Cambodian patients were classified by the local physician using the WHO classification of 1997. For the present study, patients classified DF or DHF grade I were considered as classic dengue. Patients classified DHF grade III and IV were considered as severe dengue. Clinical data were collected and included details on the platelet count, transaminases level, blood cells count, biochemistry (cholesterol, triglycerides, bilirubin…), ultrasound imagery, hemorrhagic signs (petechiae, ecchymosis, epistaxis…), the presence/or absence of warning signs (persistent vomiting, abdominal pain…), plasma leakage and shock signs (cold extremities, cyanosis…).

The main characteristics of the Cambodian patients are detailed in Table 3 (Cambodian patients). The mean age of patients from Cambodia (about 8 years-old) was lower than that of the Colombian patients. The male/female ratios of the DF and SD groups were similar. All Cambodian patients were infected with serotype 1 viruses. The plasma samples were collected approximately 3 days after the onset of symptoms (=acute samples) and also just before the discharge from the hospital (=discharge samples).

**Fig. 4:**
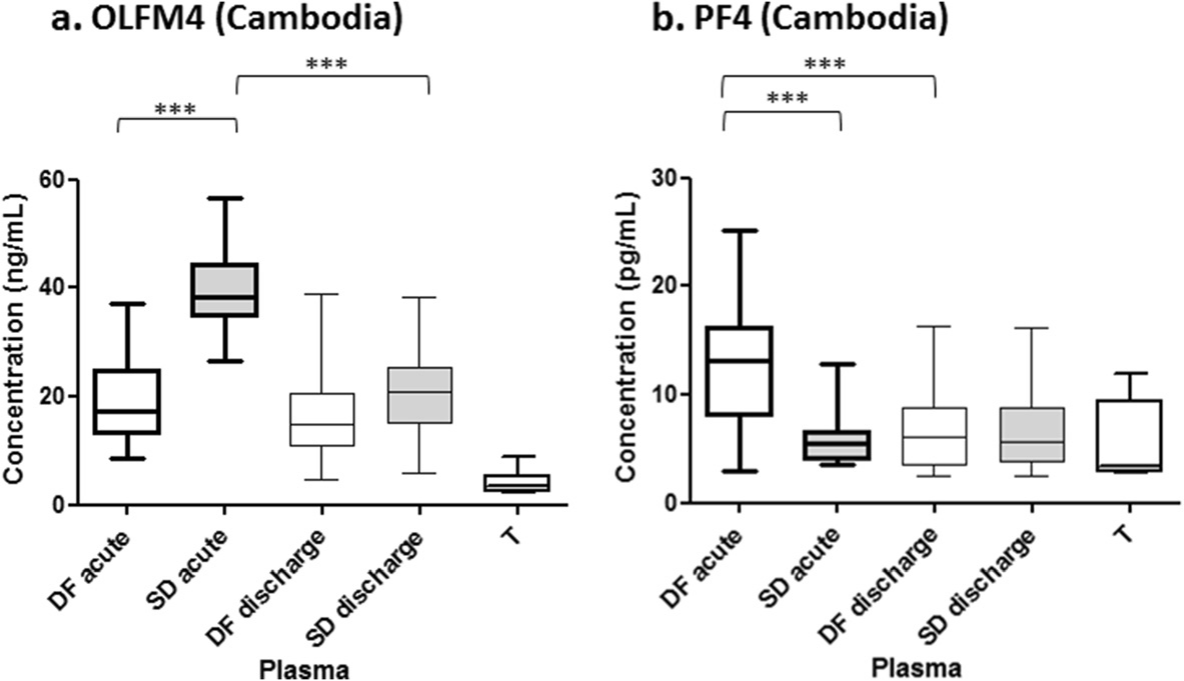
Assessment of the protein concentrations of OLFM4 (**a**) and PF4 (**b**) in the individual plasma samples of Cambodian patients using specific quantitative ELISAs. Patients were taken during both the acute phase (DF acute and SD acute) and at time of discharge from the hospital (DF discharge and SD discharge). T: healthy patient samples. Each value corresponds to the mean of two independent experiments, each performed in duplicate. **: 0.005 < *p* < 0.01; ***: *p* < 0.005

In contrast to the samples obtained from the Colombian patients, ELISAs showed that the levels of C1r and CP were not significantly different between DF and SD patients in the Cambodian cohort (see Additional file 4); however, concentrations of OLFM4 (*P* < 0.0001) and PF4 (*P* <0.0001) during the acute phase were significantly different between DF and SD plasma (Fig. 4). The OLMF4 concentration in DF patients was lower than in SD patients. In contrast, PF4 concentrations were higher in DF patients than in SD patients and SD patients showed no significant difference with healthy controls (*n* = 8).

For these two markers, at the time of discharge, concentrations tended to decline back to the levels observed in controls.

Receiver operating characteristic (ROC) curves compare sensitivity versus specificity across a range of values. ROC curves are used to assess the discriminatory ability of each markers. Area under the ROC curve (AUC) is another measure of test performance. The AUC quantifies the overall ability of the test to discriminate between individuals with DF and those with SD. A perfect test (zero false positives and zero false negatives) has an AUC of 1.00.

ROC curve analysis was used to determine AUCs and specificity/sensitivity values for OLFM4 and PF4 as prognostic biomarkers of disease severity in Cambodian acute patients with dengue. The ROC curves indicated good discrimination between DF and SD as the AUC were 0.958 for OLFM4 and 0.836 for PF4. The highest specificity/sensitivity values obtained using this model are summarized in Table 4. For a sensitivity of 96 %, the specificity exceeded 62 % (PF4) or 86 %(OLMF4). When the sensitivity reached 100 %, the specificity dropped to 56.25 % (PF4) or 82.6 % (OLFM4).

**Table 4:** Summary of ROC analysis of the prognostic value of OLFM4 and PF4 in the Cambodian cohort (*n* = 49)

	AUC (95 % CI)	For a sensitivity of:	The highest specificity is:
OLFM4	0.958 (0.906–1)	100 %	82.6 %
		96 %	86.9 %
PF4	0.836 (0.689–0.983)	100 %	56.25 %
		96 %	62.5 %

*AUC* area under the curve, *CI* confidence interval

## Discussion

Dengue viral infections are prevalent in tropical and subtropical areas, and are associated with substantial morbidity and mortality. The pathogenesis of dengue remains unclear. Various proteomic approaches have been used to characterize host-protein changes during DV infection and identify prognostic biomarkers of disease severity; several studies have been conducted on cells infected in vitro [29–31]. Studies on plasma specimens have identified a number of candidate biomarkers [32–34].

As the dynamic range for plasma proteins is large (over 10 orders of magnitude) and the most abundant proteins (0.1 % of the total number) constitute up to 95 % of the plasmatic protein mass, identification of biomarkers present at low concentrations is a major challenge in proteomic studies dealing with plasma. However, proteomic techniques are constantly being improved to assess low abundance plasma proteins [35].

Viruses facilitate their replication and propagation by subverting host cellular pathways and processes, and constantly adapt to and modulate their host’s environment. Enveloped viruses are able to incorporate numerous host proteins, both into virus particles as well as the host-derived viral envelope. As the genomes of RNA viruses only encode a small number of proteins, they must rely on host proteins during an infection. Interactions between the virus and host may have unforeseen consequences, depending on viremia level, the host’s genetic background and other relevant factors [36]. Host proteins can be incorporated into virions either randomly, by being present at the site of budding, or specifically, as a result of interacting with viral proteins.

The functional significance of the host proteins that associate with viral particles remains to be thoroughly investigated; however, it is very likely that such interactions contribute to pathogenicity if they disturb the metabolism of the host cell [37]. For instance, the incorporation of cellular proteins such as integrins or HLA class II proteins can affect the ability of HIV-1 to infect host cells and contributes to immunopathogenesis [38, 39].

Viroproteomic analyses have already been conducted on DNA viruses [40] and RNA viruses, such as retroviruses [39, 41], paramyxoviruses [42], coronaviruses [43] and flaviviruses [44]; these studies have proven that many cellular proteins are incorporated into the virion or associate with the viral membrane.

Usually, prior to MS analysis, protease hydrolysis is combined with ultracentrifugation to remove host proteins that may co-purify with virions. In the present study, as we aimed to characterize the host proteins that interact with viral membrane proteins and particles, enzymatic hydrolysis of the proteins outside of the virion could not be performed. Therefore, a new technique combining ultracentrifugation with water-insoluble polyelectrolyte-based enrichment was developed to enable proteomic characterization of a fraction of human plasma enriched with virus particles and depleted of the host proteins predominantly expressed in plasma.

Previous studies measuring viremia have demonstrated that patients with SD have higher viremia than patients with DF [9, 16, 17]. Higher viremia may lead to biosynthesis of virions with an altered host protein composition, which may reflect an increased level of cellular stress, or subtle changes in the assembly pathway. These host proteins may be packaged into the virus particle along with the viral components or incorporated into the viral membrane. Alternatively, they can be simply co-purified along with virions. Such proteins may potentially be fingerprints of the virus assembly pathway and may also play a role in viral pathogenicity.

Nano LC-MS/MS was used in this study to analyze the protein composition of the virion-enriched samples purified from the plasma pools. Some proteins had a higher average number of peptides in the SD sample than the DF and control samples, suggesting the presence of greater amounts of these proteins associated to viruses purified from the plasma of patients with SD; conversely, other proteins were mainly identified in the samples purified from the DF plasma pool.

Interestingly, the identified host proteins belong to three main pathways: the Complement System, the Coagulation System and the Acute Response Signaling Pathway. The Complement System plays an important role in both innate and adaptive immune responses, is first line of defense against infectious agents, and modulates B- and T-cell responses. In *Flavivirus* infections, excessively-activated complement proteins have been reported to induce a deleterious, exacerbated inflammatory response [45–47]. Recently, it has been shown that the level of complement proteins positively correlates with the severity of dengue in Indonesian patients [48].

Coagulation is the process by which the blood changes from a liquid to a gel. Coagulation is required to stop blood loss and enable the subsequent repair of damaged vessels. Hemorrhage is one of the major symptoms of SD, and is probably caused by vasculopathy and a deficiency in coagulation and fibrinolysis. The normal vascular endothelium produces inhibitors of coagulation and fibrinolysis. Hemostasis can be impaired if the endothelium is stimulated by excessive levels of cytokines or by a pathogen, which may in turn result in thrombosis and bleeding [49, 50].

The Acute Phase Response corresponds to the inflammatory response observed in response to an infection, tissue injury or an immunological disorder [51], and is mainly characterized by increased levels of inflammatory factors and a change in the protein composition of plasma. Interestingly, these changes can inhibit complement activation [52].

Interconnections between the coagulation process and complement cascade have been reported by many authors [53, 54]. Deregulation of one or both systems can result in the clinical manifestations of diseases with inflammatory complications. In a previous study, differentially-expressed genes associated with the immune response were identified by microarray analysis of peripheral blood mononuclear cells isolated from Colombian children with dengue fever or dengue hemorrhagic fever. These results indicated that the complement and numerous cytokines are deregulated in patients with dengue-hemorrhagic fever. Such changes may enhance disease severity by disturbing coagulation and inducing endothelial cell damage [55]. In another recent study, sera from Indian patients with DF were compared with that of healthy controls using 2D-DIGE associated with MALDI TOF/TOF MS. The authors reported that DV infection led to altered expression of multiple serum proteins involved in complement cascades, blood coagulation and acute phase response signaling, providing further clues regarding the pathogenesis and host immune response to DV infection [56].

The Complement System, Coagulation System and Acute Response Signaling Pathway are strongly interconnected and share proteins whose expression is modulated during an infection. They are all related to the inflammatory process and the innate immune response, and act upstream of activation of the adaptive immune response. Expression deregulation of these proteins could induce deleterious effects in endothelial cells [57]. In our hands, none of the seven complement proteins tested by ELISA showed a difference in concentration levels between SD and DF acute samples, minimizing the potential role of these proteins in dengue pathogenesis, at least in the early phase of the disease.

Several authors have reported proteomic analysis of samples from patients with dengue using different approaches [29, 30, 32]. To our knowledge, this study is the first assessment of the proteins that are differentially present in a virus-enriched fraction purified from plasma specimens obtained from patients during the acute stage of DV infection.

We were unable to confirm that proteins identified by LC-MS/MS were directly associated with the viral particles. Neither ELISAs conducted on virions purified from plasma, nor electron microscopy using specific gold-labeled antibodies succeed to yield reliable results. Consequently, we cannot discard the possibility that the host proteins identified in this study may be contaminants that co-purify reproducibly with viral particles.

ELISAs carried out on individual plasma specimens from the Colombian cohort indicated trends towards higher levels of CP, C1r and OLFM4 in patients with SD compared to patients with DF.

CP, an acute phase protein, is a ferroxidase involved in iron metabolism. Elevated levels of CP are generally observed during inflammation. C1r is a complement protein that interacts with C1s at the beginning of the complement cascade. OLFM4 is an anti-apoptotic factor that promotes tumor growth and facilitates cell adhesion, probably via interacting with cell surface lectins and cadherin [58]. The associations between these proteins and disease severity in dengue remain to be explored. Interestingly, a recent paper mentioned the interest of OLFM4 as a marker of disease severity in Respiratory Syncytial Virus infection in children [59].

ELISAs also showed that the concentration of PF4 was higher in the plasma samples of Colombian patients with DF than patients with SD. PF4 is a small cytokine released by platelets that promotes blood coagulation by moderating the effects of heparin-like molecules. PF4 also plays a role in wound repair and inflammation. Alterations in platelet function have been associated with plasma leakage, which is one of the major features of severe disease in patients with dengue [60]. Recently, it has been demonstrated that DV replicates and produces infectious virus in platelets [61]. In 1989, Srichaikul T. et al. demonstrated that the level of PF4 increases during acute phase in both shock or non-shock DHF children. It is difficult to compare these results with the results presented here because there is no strict correspondence between the patient classification used by Srichaikul T. et al. and the classification of 1997 used in the present work [62]. Interestingly, among the six proteins involved in coagulation and having a SD/DF peptide ratio higher than 1.3 or lower than 0.78 (see Additional file 1), PF4 is the only one that was confirmed by ELISA. PF4 is significantly less concentrated in SD acute patient plasma.

The trends observed for OLFM4 and PF4 by ELISAs for the Colombian patients were also confirmed in the Cambodian patients. By testing different time points during the course of the disease, we showed that these markers evolved through time and tended, during the process of healing, to get close to the protein concentration found in healthy patients. PF4 was overabundant in DF acute patients, but remained surprisingly low in SD patients, with levels remaining similar to control individuals. It has previously been proposed that PF4 interacts with the vasculature and is involved in thrombus formation at sites of vascular injury [63]. Murine studies suggest that PF4 may have an overall salutary effect in sepsis [64]. Therefore, a basal level of PF4 expression during the acute phase of DV infection could be an interesting marker of a future severe dengue.

The severity of dengue is modulated by multiple risk factors such as the age, genetic background and nutritional status of the host, as well as the genotype and serotype of the virus. These factors could explain the differences in the concentrations of specific markers observed between the plasma samples from the Colombian and Cambodian patients. For example, SD in South-East Asia is mainly observed in children, whereas adults are predominantly affected in South America [65]. As a consequence, results should be extrapolated with caution to different geographic areas or different demographic groups.

## Conclusion

In conclusion, we describe the development of a novel technique of DV-enrichment from complex biological fluids based on centrifugation combined with a water-insoluble polyelectrolyte-based technique, for subsequent proteomic analyses. We found no evidence that the identified host proteins are specifically associated with virions. However, this purification technique enables the analysis of a plasma fraction enriched in virions and from which the most abundant plasma proteins were removed. The host proteins characterized in this study may potentially reflect how DV infection disturbs the function of the cellular proteome. In this regard, further studies are required to assess the prognostic value of host proteins associated with inflammation, complement cascade and coagulation for disease severity by analysis of additional biological samples from patients infected with DV.

Analysis of a selection of the identified proteins using ELISAs identified two host proteins, OLFM4 and PF4, which had significant prognostic value for classifying patients with dengue who were likely to develop SD. Further prospective studies are warranted to confirm and validate the prognostic value of OLFM4 and PF4 as potential biomarkers of disease severity in larger cohorts of patients from a variety of dengue-endemic areas around the globe.

## Availability of data and materials

Data supporting the findings and materials are available upon request to Frederic Bedin, BioMerieux SA, Chemin de l’Orme, 69280 Marcy l’Etoile (France).

## Additional files

Additional file 1:
**Virus-enriched fraction proteome change in purified plasma pools obtained from acute dengue patients.** (PDF 196 kb) Additional file 2:
**Expression levels and relationship of the proteins identified by LC-MS/MS in the Acute Phase Response Signaling (2), the Complement system (3) and the Coagulation system (4).** Diagrams have been obtained using the IPA software. Proteins are displayed by various shapes that represent the functional classes of proteins. Proteins in red correspond to proteins found over-represented in the SD pool. Proteins in green correspond to proteins found over-represented in the DF pool. Proteins in grey are only identified in the SD pool. The color intensity of each node is related to the level of expression. Uncolored node: no data available. (ZIP 815 kb) Additional file 3:
**Network of the proteins identified by LC-MS/MS.** The nature of the relationship between proteins is indicated by various line styles. Proteins are displayed by shapes that represent the functional classes of proteins. Proteins in red correspond to proteins found over-represented in the SD pool. Proteins in green correspond to proteins found over-represented in the DF pool. Proteins in grey are only identified in the SD pool. The color intensity of each node is related to the level of expression. (TIF 441 kb) Additional file 4:
**ELISA C1R and ceruloplasmin on DF of SD Cambodian plasma specimen.** Results showed no significant difference between the two populations tested. (TIF 65 kb)

## Abbreviations

AUC: Area Under the Curve
C1R: Complement factor 1R
CI: Confidence Interval
CP: ceruloplasmin
DF: Dengue Fever
DIGE: Difference Gel Electrophoresis
DV: Dengue Virus
ELISA: Enzyme Linked ImmunoSorbent Assay
ER: Endoplasmic Reticulum
IU: International Unit
LC-MS/MS: Liquid Chromatography coupled to Mass Spectrometry chain
MES: 2-(N-Morpholino)EthaneSulfonic acid
MOI: Multiplicity of Infection
NA: not applicable
NS: Non-Structural
OLFM4: olfactomedin-4
PAGE: PolyAcrylamide Gel Electrophoresis
PBS: Phosphate Buffer Saline
PF4: Platelet Factor-4
qRT-PCR: quantitative Reverse-Transcriptase Polymerase Chain Reaction
RNA: RiboNucleic Acids
ROC: Receiving Operating Curve
SD: Severe Dengue
SDS: Sodium Dodecyl Sulfate
