# Supplementary material for: Differential proteomic analysis of virus-enriched fractions obtained from plasma pools of patients with dengue fever or severe dengue
Source: BMC Infect Dis. 2015 Nov 14;15:518. doi: 10.1186/s12879-015-1271-7 (PMC4647599; doi:10.1186/s12879-015-1271-7)
Supplement: Additional file 1: — Virus-enriched fraction proteome change in purified plasma pools obtained from acute dengue patients. (PDF 196 kb) [file 12879_2015_1271_MOESM1_ESM.pdf]

S1

Accession number	Protein name	Average DF peptide	Average SD peptide	RatioSD/DF peptide
P09871	Complement C1s subcomponent (C1 esterase)	3,5	14,5	4,14
P07225	Vitamin K-dependent protein S	1	4	4,00
P01008	Antithrombin-III (ATIII) (Serpin C1)	3	9,5	3,17
Q16610	Extracellular matrix protein 1 (Secretory component p85)	1	3	3,00
P27918	Properdin (Complement factor P)	1,5	4	2,67
B4E1B2	Serotransferrin	11	29	2,64
Q53H26	Transferrin variant (Fragment)	11	29	2,64
B4DPQ0	Complement C1r subcomponent	5	13	2,60
P01019	Angiotensinogen (Serpin A8)	1	2,5	2,50
B4DDU2	Tubulin alpha-ubiquitous chain	2,5	6	2,40
P01779	Ig heavy chain V-III region TUR	2,5	6	2,40
P68366	Tubulin alpha-4A chain (Alpha-tubulin 1)	3	6,5	2,17
P00734	Prothrombin (Coagulation factor II)	2	4	2,00
P21333	Filamin-A (Actin-binding protein 280)	14,5	28,5	1,97
P00450	Ceruloplasmin (EC 1.16.3.1) (Ferroxidase)	8,5	16,5	1,94
E7EX29	14-3-3 protein zeta/delta (Fragment)	4,5	8,5	1,89
P63104	14-3-3 protein zeta/delta (KCIP-1)	4,5	8,5	1,89
B4E1D8	cDNA FLJ51597, highly similar to C4b-binding protein	10,5	19	1,81
P18206	Vinculin (Metavinculin)	5	9	1,80
P12814	Alpha-actinin-1 (Alpha-actinin cytoskeletal isoform)	6,5	11,5	1,77
O43707	Alpha-actinin-4 (F-actin cross-linking protein)	6,5	11,5	1,77
P06396	Gelsolin (AGEL) (Actin-depolymerizing factor)	3,5	6	1,71
P05106	Integrin beta-3 (Platelet membrane glycoprotein IIIa) (GPIIIa)	5,5	9	1,64
P08514	Integrin alpha-IIb (GPIIb) (Platelet membrane glycoprotein IIb)	12,5	20	1,60
P01833	Polymeric immunoglobulin receptor (PIgR)	2,5	4	1,60
Q9Y490	Talin-1	28,5	43	1,51
A2J1N9	Rheumatoid factor RF-ET12 (Fragment)	3	4,5	1,50
Q5NV90	V2-17 protein (Fragment)	3	4,5	1,50
P01023	Alpha-2-macroglobulin (Alpha-2-M)	56,5	83	1,47
A2KBC6	Anti-FactorVIII scFv (Fragment)	6,5	9,5	1,46

P01011	Alpha-1-antichymotrypsin (ACT) (Serpin A3)	3,5	5	1,43
P00738	Haptoglobin	13,5	19	1,41
P02743	Serum amyloid P-component (SAP)	2,5	3,5	1,40
P01024	C3 and PZP-like alpha-2-macroglobulin domain-containing protein 1	60,5	84,5	1,40
A8K008	cDNA FLJ78387	17,5	24	1,37
A2IPI5	HRV Fab 026-VL (Fragment)	3	4	1,33
Q6UX06	Olfactomedin-4 (OLM4) (Antiapoptotic protein GW112)	4,5	6	1,33
P03952	Plasma kallikrein (EC 3.4.21.34) (Fletcher factor)	1,5	2	1,33
P08567	Pleckstrin (Platelet 47 kDa protein) (p47)	1,5	2	1,33
P07996	Thrombospondin-1	19,5	26	1,33
P07437	Tubulin beta chain (Tubulin beta-5 chain)	4,5	6	1,33
A2MYD4	V2-7 protein (Fragment)	3	4	1,33
P00488	Coagulation factor XIII A chain (EC 2.3.2.13)	3,5	4,5	1,29
Q71U36	Tubulin alpha-1A chain (Alpha-tubulin 3)	3,5	4,5	1,29
P68363	Tubulin alpha-1B chain (Alpha-tubulin ubiquitous)	3,5	4,5	1,29
Q9BQE3	Tubulin alpha-1C chain (Alpha-tubulin 6)	3,5	4,5	1,29
Q9H4B7	Tubulin beta-1 chain	3,5	4,5	1,29
Q96K68	cDNA FLJ14473 fis, clone MAMMA1001080	12	15	1,25
P08107	Heat shock 70 kDa protein 1A/1B	2	2,5	1,25
P34931	Heat shock 70 kDa protein 1-like	2	2,5	1,25
P11142	Heat shock cognate 71 kDa protein	2	2,5	1,25
Q6N089	Putative uncharacterized protein DKFZp686P15220	15	18,5	1,23
P02675	Fibrinogen beta chain	11	13,5	1,23
P00739	Haptoglobin-related protein	12	14,5	1,21
Q86UX7	Fermitin family homolog 3 (Kindlin-3)	5	6	1,20
P02751	Fibronectin (FN)	61,5	72,5	1,18
B7ZLE5	FN1 protein	61,5	72,5	1,18
Q6MZM7	Putative uncharacterized protein DKFZp686O12165	61,5	72,5	1,18
Q68CX6	Putative uncharacterized protein DKFZp686O13149	61,5	72,5	1,18
P68032	Actin, alpha cardiac muscle 1 (Alpha-cardiac actin)	8,5	10	1,18
Q562R1	Beta-actin-like protein 2 (Kappa-actin)	8,5	10	1,18
P0C0L4	Complement C4-A (Acidic complement C4)	52	61	1,17
P0C0L5	Complement C4-B (Basic complement C4)	52	61	1,17
B0UZ85	Complement component 4B (Childo blood group)	53	62	1,17

Q6MZQ6	Putative uncharacterized protein DKFZp686G11190	15	17,5	1,17
P07477	Trypsin-1 (EC 3.4.21.4) (Serine protease 1)	3	3,5	1,17
Q4TZM4	Hemoglobin beta chain (Fragment)	6,5	7,5	1,15
P04004	Vitronectin (VN) (S-protein) (V75)	6,5	7,5	1,15
B2RUT6	Complement component 4A (Rodgers blood group)	53,5	61,5	1,15
P01031	Complement C5	3,5	4	1,14
Q9UL78	Myosin-reactive immunoglobulin light chain variable region	3,5	4	1,14
Q5NV62	V3-4 protein (Fragment)	6	7	1,17
A6H8M8	C4A protein (Complement C4 gamma chain)	53,5	61	1,14
P02768	Serum albumin	51,5	58,5	1,14
Q7Z351	Putative uncharacterized protein DKFZp686N02209	15	17	1,13
P02649	Apolipoprotein E (Apo-E)	4	4,5	1,13
P01009	Alpha-1-antitrypsin (Alpha-1 protease inhibitor) (Serpin A1)	8,5	9,5	1,12
Q08380	Galectin-3-binding protein (Basement membrane autoantigen p105)	12	13	1,08
Q6N092	Putative uncharacterized protein DKFZp686K18196 (Fragment)	12	13	1,08
P63261	Actin, cytoplasmic 2 (Gamma-actin)	20	21,5	1,08
P02671	Fibrinogen alpha chain	9,5	10	1,05
P60709	Actin, cytoplasmic 1 (Beta-actin)	19,5	20,5	1,05
A2MYE1	A30 (Fragment)	4	4	1,00
Q96SA9	Anti-streptococcal/anti-myosin immunoglobulin kappa light chain	3,5	3,5	1,00
P06576	ATP synthase subunit beta, mitochondrial (EC 3.6.3.14)	2	2	1,00
O43866	CD5 antigen-like (CT-2) (IgM-associated peptide)	15	15	1,00
P12259	Coagulation factor V (Activated protein C cofactor)	1,5	1,5	1,00
P02747	Complement C1q subcomponent subunit C	5,5	5,5	1,00
P04196	Histidine-rich glycoprotein (HPRG)	1,5	1,5	1,00
P01892	HLA class I , A-2 alpha chain (MHC class I antigen A*2)	3	3	1,00
P30447	HLA class I histocompatibility antigen, A-23 alpha chain	3	3	1,00
F6IQP2	MHC class I antigen (Fragment)	3	3	1,00
F6IR35	MHC class I antigen (Fragment)	3	3	1,00
A2NKM7	NANUC-2 heavy chain (Fragment)	3	3	1,00
P26022	Pentraxin-related protein PTX3 (Pentaxin-related protein PTX3)	2	2	1,00
P02760	Protein AMBP	2,5	2,5	1,00
A2J1M8	Rheumatoid factor RF-IP12 (Rheumatoid factor RF-IP13)	3	3	1,00
A2J1N0	Rheumatoid factor RF-IP14 (Fragment)	6	6	1,00

Q9HCC1	Single chain Fv (Fragment)	7,5	7,5	1,00
H0YLA9	Uncharacterized protein	2	2	1,00
P02774	Vitamin D-binding protein (DBP) (VDB)	2	2	1,00
P02647	Apolipoprotein A-I (ApoA-I) (Apolipoprotein A1)	11	10,5	0,95
P68871	Hemoglobin subunit beta (Beta-globin)	8,5	8	0,94
A2NYQ9	Anti-folate binding protein (Fragment)	7	6,5	0,93
A2JA14	Anti-mucin1 heavy chain variable region (Fragment)	6,5	6	0,92
B6EDE2	Epididymis luminal protein 180 (Fragment)	6,5	6	0,92
Q14477	Hbbm fused globin protein (Fragment)	6,5	6	0,92
Q670S4	Hemoglobin Lepore-Baltimore (Fragment)	6,5	6	0,92
P02042	Hemoglobin subunit delta (Delta-globin)	6,5	6	0,92
Q9UL90	Myosin-reactive immunoglobulin heavy chain variable region	6,5	6	0,92
A2NZ55	Variable immnoglobulin anti-estradiol heavy chain	6,5	6	0,92
Q15485	Ficolin-2 (37 kDa elastin-binding protein)	5,5	5	0,91
Q9UL88	Myosin-reactive immunoglobulin heavy chain variable region	5	4,5	0,90
Q6ZVX0	cDNA FLJ41981 fis, clone SMINT2011888	9,5	8,5	0,89
Q7Z374	Putative uncharacterized protein DKFZp686C02218	9,5	8,5	0,89
Q6MZX9	Putative uncharacterized protein DKFZp686M08189	9,5	8,5	0,89
P02679	Fibrinogen gamma chain	12,5	11	0,88
Q6P5S8	antioxidant activity; very-low-density lipoprotein particle remodeling	16	14	0,88
P27105	Erythrocyte band 7 integral membrane protein (Stomatin)	4	3,5	0,88
P69905	Hemoglobin subunit alpha (Alpha-globin)	4	3,5	0,88
Q6GMX0	Putative uncharacterized protein	16	14	0,88
P02792	Ferritin light chain (Ferritin L subunit)	3,5	3	0,86
Q9UL71	Myosin-reactive immunoglobulin heavy chain variable region	7	6	0,86
Q07954	Prolow-density lipoprotein receptor-related protein 1 (LRP-1)	7	6	0,86
A1A508	PRSS3 protein	3,5	3	0,86
Q6P5S3	Putative uncharacterized protein	7	6	0,86
Q65ZC9	Single-chain Fv (Fragment)	7	6	0,86
Q5CZ94	Putative uncharacterized protein DKFZp781M0386	6,5	5,5	0,85
P19823	Inter-alpha-trypsin inhibitor heavy chain H2 (ITI-HC2)	9,5	8	0,84
Q16195	Keratin (Fragment)	9,5	8	0,84
P30464	HLA class I , B-15 alpha chain (MHC class I antigen B*15)	3	2,5	0,83
P03989	HLA class I , B-27 alpha chain (MHC class I antigen B*27)	3	2,5	0,83

Q9UL83	Myosin-reactive immunoglobulin light chain variable region	3	2,5	0,83
P35579	Myosin-9 (Myosin heavy chain 9)	8,5	7	0,82
A0A5E4	Putative uncharacterized protein	8,5	7	0,82
P10909	Clusterin (Apolipoprotein J) (Apo-J)	5,5	4,5	0,82
Q5EFE6	Anti-RhD monoclonal T125 kappa light chain	16	13	0,81
Q86TT1	Full-length cDNA clone CS0DD006YL02 of Neuroblastoma	28	22,5	0,80
O14791	Apolipoprotein L1 (ApoL-I)	2,5	2	0,80
A2NB45	Cold agglutinin FS-1 L-chain (Fragment)	2,5	2	0,80
A2NB44	Cold agglutinin FS-2 H-chain (Fragment)	2,5	2	0,80
B1N7B8	Cryocrystalglobulin CC1 kappa light chain variable region	2,5	2	0,80
Q86SX2	Full-length cDNA clone CS0DL004YM19 of B cells	2,5	2	0,80
Q7Z3Y6	Rearranged VH4-34 V gene segment (Fragment)	2,5	2	0,80
A0N5G5	Rheumatoid factor D5 light chain (Fragment)	2,5	2	0,80
H0YK52	Uncharacterized protein	2,5	2	0,80
Q14624	Inter-alpha-trypsin inhibitor heavy chain H4 (ITI-HC4)	16	12,5	0,78
P31946	14-3-3 protein beta/alpha (KCIP-1)	4,5	3,5	0,78
P62258	14-3-3 protein epsilon (14-3-3E)	4,5	3,5	0,78
Q04917	14-3-3 protein eta (Protein AS1)	4,5	3,5	0,78
P61981	14-3-3 protein gamma (KCIP-1)	4,5	3,5	0,78
P27348	14-3-3 protein theta (Protein HS1)	4,5	3,5	0,78
A2NUT2	Lambda-chain (AA -20 to 215)	9	7	0,78
P02776	Platelet factor 4 (PF-4)	4	3	0,75
P10720	Platelet factor 4 variant (CXCL4L1)	4	3	0,75
P02746	Complement C1q subcomponent subunit B	9,5	7	0,74
P48740	Mannan-binding lectin serine protease 1 (EC 3.4.21.-)	7,5	5,5	0,73
Q96JD0	Amyloid lambda 6 light chain variable region SAR (Fragment)	9	6,5	0,72
Q6GMV8	Putative uncharacterized protein	9	6,5	0,72
Q8NEJ1	Putative uncharacterized protein	9	6,5	0,72
A0N5G3	Rheumatoid factor G9 light chain (Fragment)	9	6,5	0,72
P02745	Complement C1q subcomponent subunit A	3,5	2,5	0,71
P60660	Myosin light polypeptide 6 (MLC-3)	3,5	2,5	0,71
Q6MZU6	Putative uncharacterized protein DKFZp686C15213	17,5	12,5	0,71
Q6N093	Putative uncharacterized protein DKFZp686I04196 (Fragment)	17,5	12,5	0,71
P61224	Ras-related protein Rap-1b (GTP-binding protein smg p21B)	3	2	0,67

P19105	Myosin regulatory light chain 12A (MLC-2B)	4	2,5	0,63
P05387	60S acidic ribosomal protein P2 (Renal carcinoma antigen NY-REN-44)	2,5	1,5	0,60
P19338	Nucleolin (Protein C23)	10	6	0,60
O75636	Ficolin-3 (Collagen/fibrinogen domain-containing lectin 3 p35)	3,5	2	0,57
F8VWA4	Uncharacterized protein	3,5	2	0,57
B7Z539	cDNA FLJ56954	8	4,5	0,56
Q96CX2	BTB/POZ domain-containing protein KCTD12 (Pfetin)	4,5	2,5	0,56
P02730	Band 3 anion transport protein(AE 1)	17,5	9,5	0,54
P23528	Cofilin-1 (p18)	3	1,5	0,50
P06748	Nucleophosmin (NPM)	3	1,5	0,50
Q6N091	Putative uncharacterized protein DKFZp686C02220	7	3,5	0,50
Q6N041	Putative uncharacterized protein DKFZp686O16217 (Fragment)	7	3,5	0,50
P11166	Solute carrier family 2, facilitated glucose transporter member 1	2	1	0,50
P04275	von Willebrand antigen 2	61,5	28,5	0,46
P62805	Histone H4	2,5	1	0,40
Q13201	Multimerin-1 (EMILIN-4)	11	4	0,36
B7Z1F8	cDNA FLJ53025, highly similar to Complement C4-B	52	16	0,31
P04114	Apolipoprotein B-100 (Apo B-100)	25	7,5	0,30
Q8TCG3	TPMsk3 (Fragment)	5	1,5	0,30
P16452	Erythrocyte membrane protein band 4.2 (P4.2)	5,5	1	0,18
P11277	Spectrin beta chain, erythrocyte (Beta-I spectrin)	23	3	0,13
P02549	Spectrin alpha chain, erythrocyte (Erythroid alpha-spectrin)	33	1,5	0,05

**Accession number**	**protein name**	**SD Average Peptides (no DF peptides)**
P00751	Complement factor B (C3/C5 convertase)	4.5
P00752	Complement component C8 alpha chain	2
P00753	Peroxiredoxin-1 (Natural killer cell-enhancing factor A) (NKEF-A)	2
P00754	Moesin (Membrane-organizing extension spike protein)	2
P00755	Chloride intracellular channel protein 1 (Chloride channel ABP)	2
P00756	Ferritin heavy chain (Ferritin H subunit)	2
