# Supplementary figures and images for: Differential proteomic analysis of virus-enriched fractions obtained from plasma pools of patients with dengue fever or severe dengue
Source: BMC Infect Dis. 2015 Nov 14;15:518. doi: 10.1186/s12879-015-1271-7 (PMC4647599; doi:10.1186/s12879-015-1271-7)

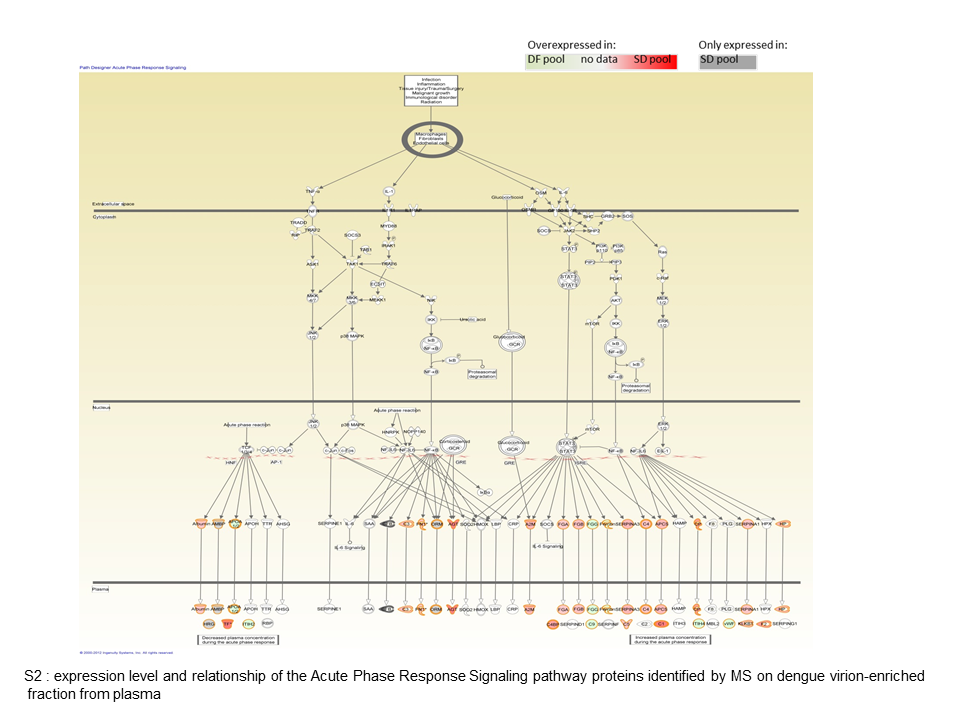

Supplement: Additional file 2: — Expression levels and relationship of the proteins identified by LC-MS/MS in the Acute Phase Response Signaling (2), the Complement system (3) and the Coagulation system (4). Diagrams have been obtained using the IPA software. Proteins are displayed by various shapes that represent the functional classes of proteins. Proteins in red correspond to proteins found over-represented in the SD pool. Proteins in green correspond to proteins found over-represented in the DF pool. Proteins in grey are only identified in the SD pool. The color intensity of each node is related to the level of expression. Uncolored node: no data available. (ZIP 815 kb) [file 12879_2015_1271_MOESM2_ESM.zip › 2R2.tif]

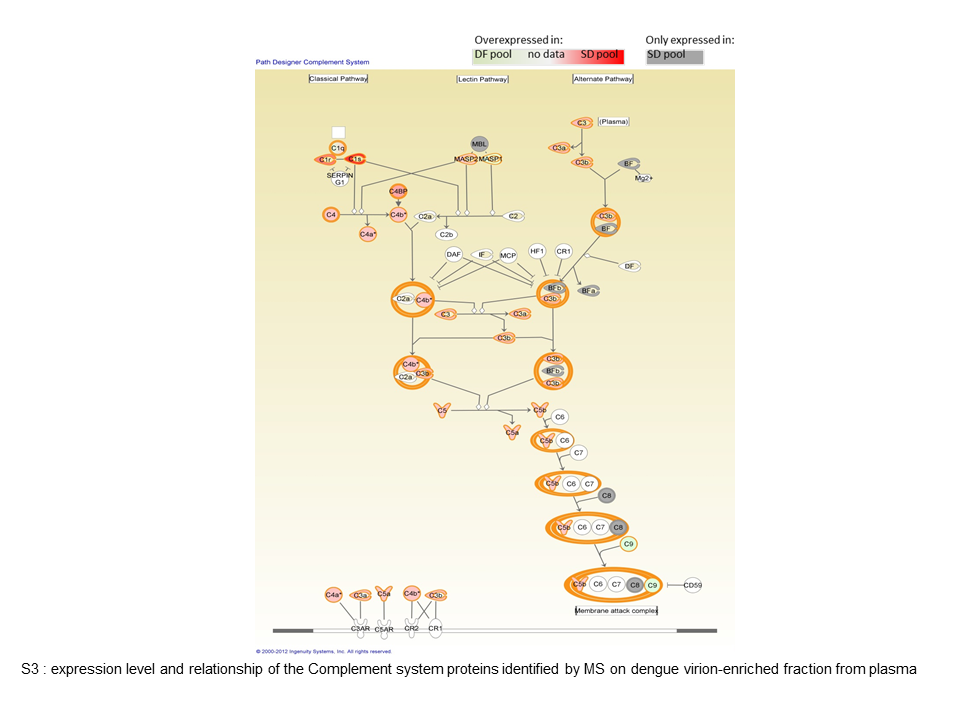

Supplement: Additional file 2: — Expression levels and relationship of the proteins identified by LC-MS/MS in the Acute Phase Response Signaling (2), the Complement system (3) and the Coagulation system (4). Diagrams have been obtained using the IPA software. Proteins are displayed by various shapes that represent the functional classes of proteins. Proteins in red correspond to proteins found over-represented in the SD pool. Proteins in green correspond to proteins found over-represented in the DF pool. Proteins in grey are only identified in the SD pool. The color intensity of each node is related to the level of expression. Uncolored node: no data available. (ZIP 815 kb) [file 12879_2015_1271_MOESM2_ESM.zip › 3R2.tif]

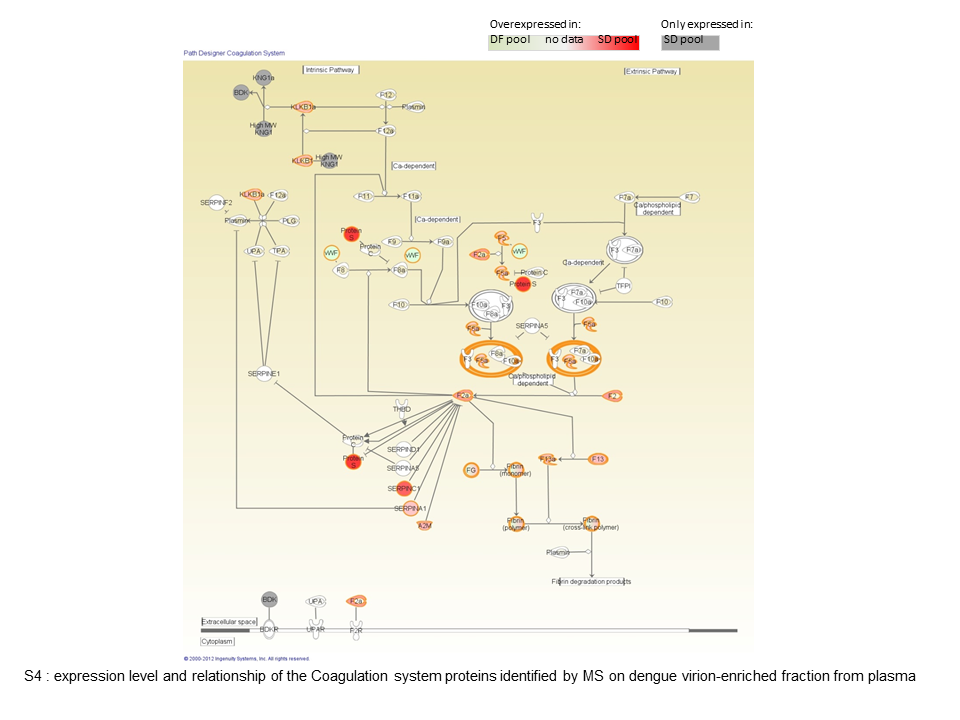

Supplement: Additional file 2: — Expression levels and relationship of the proteins identified by LC-MS/MS in the Acute Phase Response Signaling (2), the Complement system (3) and the Coagulation system (4). Diagrams have been obtained using the IPA software. Proteins are displayed by various shapes that represent the functional classes of proteins. Proteins in red correspond to proteins found over-represented in the SD pool. Proteins in green correspond to proteins found over-represented in the DF pool. Proteins in grey are only identified in the SD pool. The color intensity of each node is related to the level of expression. Uncolored node: no data available. (ZIP 815 kb) [file 12879_2015_1271_MOESM2_ESM.zip › 4R2.tif]

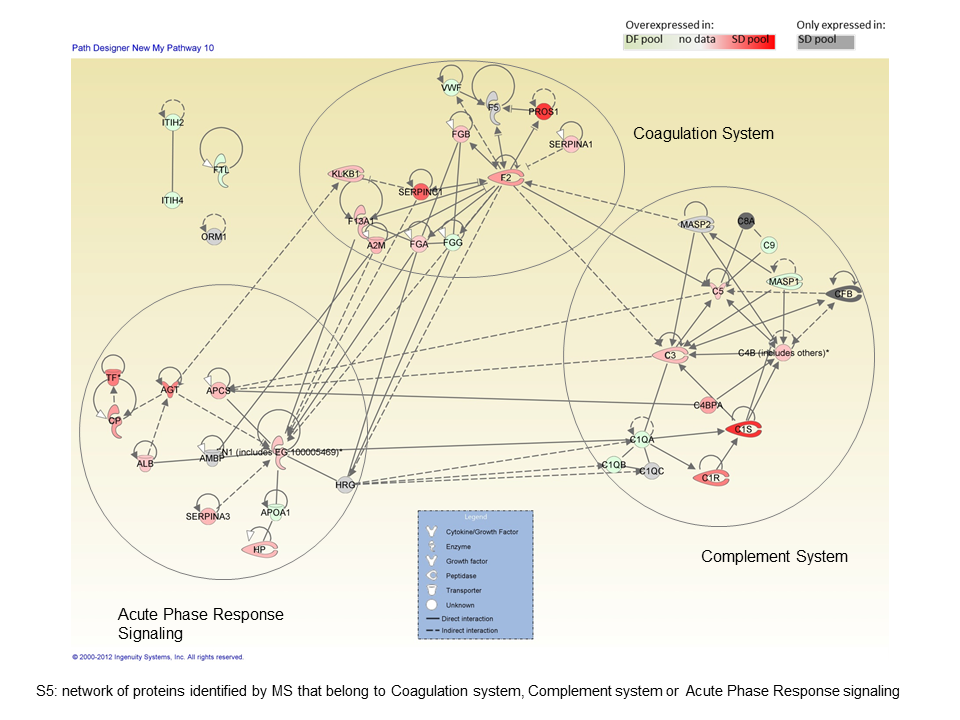

Supplement: Additional file 3: — Network of the proteins identified by LC-MS/MS. The nature of the relationship between proteins is indicated by various line styles. Proteins are displayed by shapes that represent the functional classes of proteins. Proteins in red correspond to proteins found over-represented in the SD pool. Proteins in green correspond to proteins found over-represented in the DF pool. Proteins in grey are only identified in the SD pool. The color intensity of each node is related to the level of expression. (TIF 441 kb) [file 12879_2015_1271_MOESM3_ESM.tif]

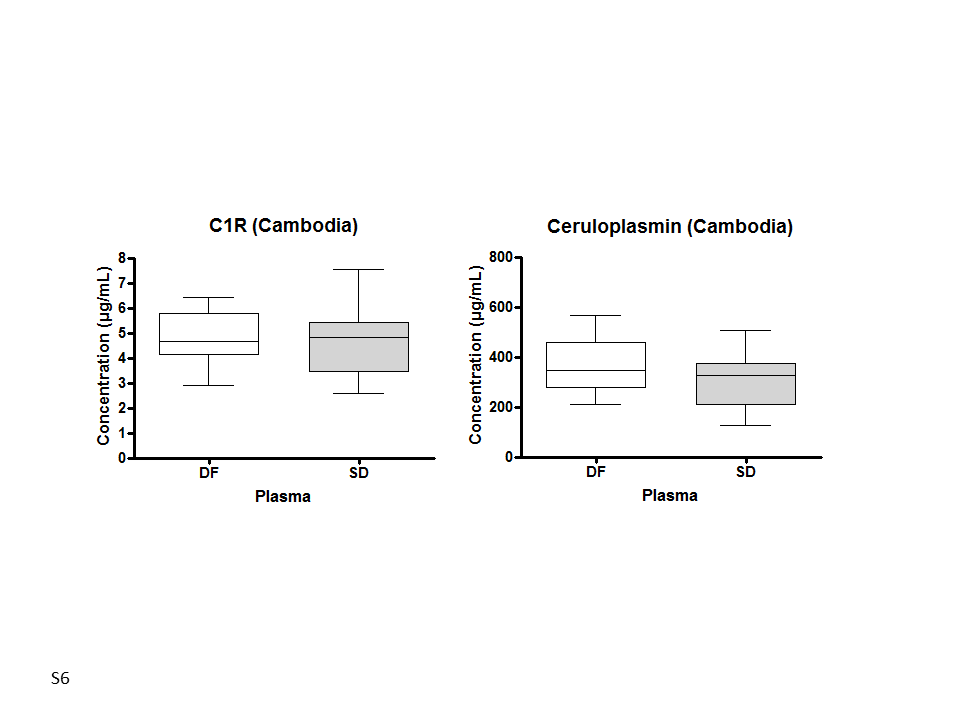

Supplement: Additional file 4: — ELISA C1R and ceruloplasmin on DF of SD Cambodian plasma specimen. Results showed no significant difference between the two populations tested. (TIF 65 kb) [file 12879_2015_1271_MOESM4_ESM.tif]
